# *Methylobacterium extorquens* RSH Enzyme Synthesizes (p)ppGpp and pppApp *in vitro* and *in vivo*, and Leads to Discovery of pppApp Synthesis in *Escherichia coli*

**DOI:** 10.3389/fmicb.2019.00859

**Published:** 2019-04-24

**Authors:** Michał Sobala, Bożena Bruhn-Olszewska, Michael Cashel, Katarzyna Potrykus

**Affiliations:** ^1^Department of Bacterial Molecular Genetics, Faculty of Biology, University of Gdańsk, Gdańsk, Poland; ^2^Intramural Program, Eunice Kennedy Shriver Institute of Child Health and Human Development, National Institutes of Health, Bethesda, MD, United States

**Keywords:** (p)ppGpp, pppApp, Rel-SpoT-homologs, stringent response, *Methylobacterium extorquens*, *Escherichia coli*

## Abstract

In bacteria, the so-called stringent response is responsible for adaptation to changing environmental conditions. This response is mediated by guanosine derivatives [(p)ppGpp], synthesized by either large mono-functional RelA or bi-functional SpoT (synthesis and hydrolysis) enzymes in β*-* and γ*-*proteobacteria, such as *Escherichia coli*. In Firmicutes and α*-*, δ*-*, and 𝜀*-*proteobacteria, large bifunctional Rel-SpoT-homologs (RSH), often accompanied by small (p)ppGpp synthetases and/or hydrolases devoid of regulatory domains, are responsible for (p)ppGpp turnover. Here, we report on surprising *in vitro* and *in vivo* properties of an RSH enzyme from *Methylobacterium extorquens* (RSH_Mex_). We find that this enzyme possesses some unique features, e.g., it requires cobalt cations for the most efficient (p)ppGpp synthesis, in contrast to all other known specific (p)ppGpp synthetases that require Mg^2+^. In addition, it can synthesize pppApp, which has not been demonstrated *in vitro* for any Rel/SpoT/RSH enzyme so far. *In vivo*, our studies also show that RSH_Mex_ is active in *Escherichia coli* cells, as it can complement *E. coli* ppGpp^0^ growth defects and affects *rrnB* P1-*lacZ* fusion activity in a way expected for an RSH enzyme. These studies also led us to discover pppApp synthesis in wild type *E. coli* cells (not carrying the RSH_Mex_ enzyme), which to our knowledge has not been demonstrated ever before. In the light of our recent discovery that pppApp directly regulates *E. coli* RNAP transcription *in vitro* in a manner opposite to (p)ppGpp, this leads to a possibility that pppApp is a new member of the nucleotide second-messenger family that is widely present in bacterial species.

## Introduction

In nature, bacteria are almost constantly faced with rapidly changing growth conditions that have led to evolution of complex and interconnected regulatory systems involving stress-specific sensing and responses. One of them is the stringent response, which was first characterized in *Escherichia coli* as a response to the onset of amino acid starvation (Cashel and Gallant, 1969; Cashel et al., 1996). This term is now generalized to include all cellular responses to virtually any environmental stress (e.g., carbon, nitrogen, phosphate, iron and lipid limitation; heat shock, and osmotic stress) that induce synthesis of specific guanosine derivatives: guanosine 5′-triphosphate-3′diphosphate (pppGpp) and guanosine 3′, 5′-bis(diphosphate) (ppGpp), collectively referred to as (p)ppGpp (Potrykus and Cashel, 2008). This response also occurs in plants (Braeken et al., 2006; Tozawa and Nomura, 2011; Field, 2018), but not in the archaea or animal kingdoms.

The hallmark of bacterial stringent response is inhibition of ribosomal RNA and tRNA synthesis with concomitant activation of stress survival gene expression, for example enhanced transcription of amino acid biosynthesis genes to cope with amino acid starvation (Cashel et al., 1996; Potrykus and Cashel, 2008). The onset of stringent response is also required for virulence of many bacterial pathogens, consistent with combatting the stress of host defense against bacterial invasion (Dalebroux et al., 2010).

In *E. coli*, and other γ- as well as β-proteobacteria, (p)ppGpp synthesis is catalyzed by two enzymes – RelA, activated by amino acid deprivation, and SpoT, activated by other environmental stresses (Potrykus and Cashel, 2008; Atkinson et al., 2011). SpoT also possesses another important function, i.e., it can hydrolyze (p)ppGpp, allowing bacteria to quickly adapt when environmental conditions are brought back to normal (Cashel et al., 1996; Potrykus and Cashel, 2008). These two enzymes are of similar length, however, RelA has lost the ability to hydrolyze (p)ppGpp. In other bacteria, such as α–, δ-, and 𝜀- proteobacteria, as well as Firmicutes, only one homologous enzyme exists; it is always bi-functional (i.e., has both, synthesis and hydrolysis activities) and such enzymes are often referred to as RSH (for Rel/SpoT Homolog). Bioinformatics analyses indicate that RSH is frequently accompanied by one or more shorter enzymes, i.e., SAS (small alarmone synthetase) or SAH (small alarmone hydrolase), each devoid of a large regulatory domain present in RelA, SpoT, and RSH (reviewed in: Atkinson et al., 2011; Steinchen and Bange, 2016).

Synthesis of (p)ppGpp generally involves transfer of the βγ-pyrophosphate from the donor ATP onto the ribose 3′ hydroxyl residue of either GTP or GDP as acceptor nucleotides, resulting in pppGpp or ppGpp, respectively (Cashel and Kalbacher, 1970). Upon hydrolysis, the same (p)ppGpp pyrophosphate residue is removed from the 3′ ribose, to yield GTP or GDP, respectively.

Understanding of the function and structure of SAS and *E. coli* RelA proteins is much advanced (Gaca et al., 2015; Steinchen et al., 2015, 2018; Beljantseva et al., 2017; Kudrin et al., 2018; Manav et al., 2018; Winther et al., 2018). In contrast, although the physiological function of full-length bi-functional RSH enzymes has been studied in several species, such as *Bacillus spp*., *Enterococcus faecalis, Deinococcus radiodurans*, and *Staphylococcus aureus* (Wendrich et al., 2000; Geiger et al., 2010; Gaca et al., 2013; Kim et al., 2014; Wang et al., 2016), their biochemical properties remain less well explored. Notable exceptions are *Streptococcus equisimilis* Rel_Seq_ (Mechold et al., 2002; Hogg et al., 2004), *Mycobacterium tuberculosis* Rel_Mtb_ (Avarbock et al., 2005; Singal et al., 2017), and *S. aureus* Rel_Sau_ (Gratani et al., 2018).

Interestingly, (p)ppGpp are not the only 3′pyrophosphate nucleotide derivatives found in bacteria. In the early work on the stringent response, (p)ppApp were discovered to be produced along with (p)ppGpp in *Bacillus subtilis* in response to addition of amino acid analogs and during sporulation, but this finding has not been pursued since (Rhaese et al., 1977; Nishino et al., 1979). So far, the only enzyme demonstrated to have such ability *in vitro* is a promiscuous pyrophosphotransferase secreted by *Streptomyces morookaensis* cells, capable of the βγ -pyrophosphate transfer from ATP or GTP onto the ribosyl-3′ hydroxyl group of any purine nucleotide (Oki et al., 1975).

We believe pppApp is worthy of further attention because recently we have demonstrated that pppApp regulates *E. coli* ribosomal promoter (*rrnB* P1) transcription *in vitro*, where contrary to (p)ppGpp mediated inhibition, transcriptional activation is observed (Bruhn-Olszewska et al., 2018). We had also shown that (p)ppApp binds near the catalytic center of *E. coli* RNA polymerase at a site distinct from (p)ppGpp site 2 (Bruhn-Olszewska et al., 2018). This suggests a regulatory role for (p)ppApp nucleotides, although their synthesis in *E. coli* has not been demonstrated until now.

Our immediate goal here is to rigorously substantiate and explore whether the catalytic domain of a previously uncharacterized RSH enzyme from *M. extorquens* (RSH_Mex_) is capable of synthesizing (p)ppGpp and/or pppApp *in vitro*. This organism was chosen as a possible source for (p)ppApp synthesis because a bioinformatics search revealed a strong homology between its single SAH enzyme and eukaryotic Mesh enzymes, which we discovered to hydrolyze (p)ppApp *in vitro* in addition to their published cleavage activity toward (p)ppGpp (Potrykus et al., unpublished). The ensuing association described here for (p)ppApp and (p)ppGpp synthetic activities of the RSH_Mex_ catalytic domain led to observations that expression of full-length RSH_Mex_ supports accumulation of both nucleotides in *E. coli* cells. Controls using wild type *E. coli* cells lacking RSH_Mex_, led to the surprising discovery that basal levels of pppApp are observed in wild type *E. coli*.

## Results

### Identification and Purification of a Putative *M. extorquens* AM1 RSH Enzyme

**FIGURE 1:**
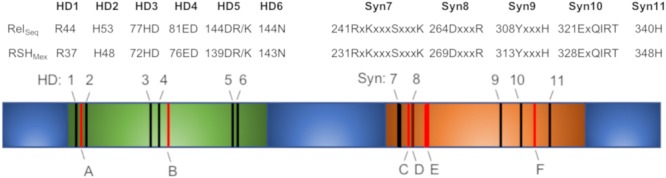
A schematic representation of RSH_Mex_ NTD domain, containing the synthetase, and hydrolase domains (RSH_Mex_1-352). The CTD regulatory domain has been omitted. Hydrolase domain (green) and synthetase domain (orange) are indicated. Black lines, HD 1-6 and Syn7-11, conserved hydrolase and synthetase motifs, respectively, as reported in (Steinchen and Bange, 2016). Corresponding Rel_Seq_ and RSH_Mex_ sequences are denoted. No deviations from the conserved sequence motifs were detected in RSH_Mex_. Red lines and **A**–**F**, previously identified in Rel_Seq_ as crucial for that enzyme’s optimal activity that are different in RSH_Mex_. **A**- 50Y/F; **B**- 97V/I; **C**- 262I/L; **D**- 267A/G; **E**- 270C/V, and 272M/V; **F**- 333H/D (numbering based on Rel_Seq_ sequence). Supplementary Figure S1 compares full length sequences of RSH_Mex_, Rel_Seq_, SpoT, and RelA.

Using DELTA-BLAST (NCBI) and the RelSeq amino acid sequence, we identified a gene in the genome of *M. extorquens* strain AM1 (GenBank accession # ACS41145.1), termed here *rsh_Mex_*, encoding a potential RSH_Mex_ protein. For enhanced clarity, we decided to use the abbreviation RSH_Mex_ rather than Rel_Mex_ suggested by (Atkinson et al., 2011), as we feel this better reflects its properties (see section “Discussion”). Comparison of RSH_Mex_ to Rel_Seq_, *E. coli* RelA and SpoT amino acid sequences using UGENE software with MUSCLE algorithm revealed that full-length RSH_Mex_ has only 38, 32, and 39% identity with those proteins, respectively (Supplementary Figure S1). However, the conserved hydrolase and synthetase domains present in the NTD domain show a more striking homology (Figure 1). Namely, all the canonical (p)ppGpp hydrolase and synthetase motifs [reviewed in (Steinchen and Bange, 2016)] are preserved. Still, several residues previously identified as crucial for optimal activity of Rel_Seq_ are different in RSH_Mex_ (Figure 1).

The DNA fragment encoding the N-terminal catalytic half of the RSH_Mex_ protein (containing the hydrolase and synthetase domains but lacking the regulatory domains) was cloned in the pCIOX expression vector for purification in the *E. coli* system. We have chosen to use the RSH_Mex_ catalytic half protein for biochemical studies and the full length protein for cellular experiments. This is because classical studies of catalytic and structural features of N-terminal half of Rel*Seq* have provided the basis for understanding general RSH features and so far, there has not been a demonstration that substrate specificity of full length RSH enzymes differs from the catalytic half protein. The added advantage is that this experimental system is simplified because ribosomes, mRNA, and uncharged tRNA are not required for either of bifunctional (synthesis or hydrolysis) activities (Mechold et al., 2002; Hogg et al., 2004). In addition, the catalytic fragment that we used (RSH_Mex_1-352) was designed to allow for direct comparison with the Rel_Seq_ studies just mentioned. Thus, all of the subsequent *in vitro* studies presented here were carried out with RSH_Mex_1-352; for employment in several control reactions, Rel_Seq_1-385 was cloned and purified in the same way as RSH_Mex_1-352.

### pppGpp Synthesis by RSH_Mex_1-352 Requires Co^2+^ for Best Efficiency

**FIGURE 2:**
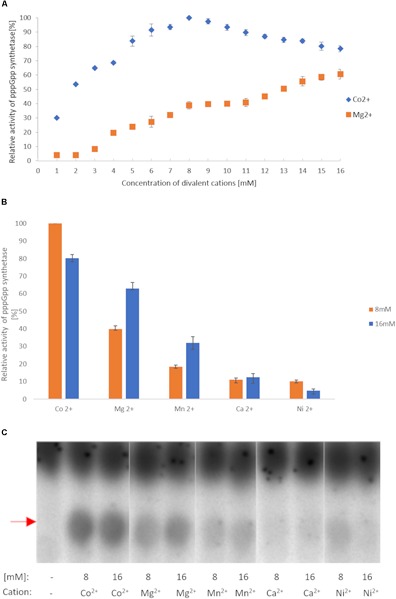
RSHMex pppGpp synthesis activity in the presence of different cations. **(A)** Mg^2+^ and Co^2+^ titration. **(B)** 8 and 16 mM concentrations of the indicated cations were used. In both cases, the reaction mixtures contained 3.3 nM [P^33^] ɣ-ATP, 8 mM unlabeled ATP, 8 mM GTP, 50 mM Tris–HCl (pH 8.9), 80 nM RSH_Mex_1-352, and were incubated for 2 h at 37°C. The relative amount of synthesized pppGpp was determined by TLC followed by densitometry. Error bars represent SD calculated from three independent experiments. **(C)** TLC separation of reactions obtained in **(B)**. Red arrow indicates pppGpp.

We first investigated (p)ppGpp synthesis by RSH_Mex_1-352 since this activity is so far present in all RSH enzymes. The initial reaction conditions chosen were similar to those previously established for Rel_Seq_1-385 (Mechold et al., 2002): 160 nM enzyme, 8 mM ATP, and 8 mM GTP were incubated at 37°C for 2 h with increasing concentrations of MgCl_2_; 3.3 nM [P^33^] γ-ATP was used as the source of label. Under these conditions, a spot corresponding to pppGpp was observed on TLC plate autoradiograms, with the highest synthesis efficiency at 16 mM Mg^2+^ (Figure 2A and Supplementary Figure S2). This agrees with Rel_Seq_ data, where it was observed that the reaction optimum is reached when the total nucleotide substrate concentration [(ATP) + (GTP)] equals that of Mg^2+^ (Mechold et al., 2002).

Next, we tested other cations at 16 mM, and found that Co^2+^ yields even higher RSH_Mex_1-352 activity (1.3 fold more pppGpp), while Mn^2+^, Ca^2+^, and Ni^2+^ are much less efficient (2, 5, and 12.8 fold less pppGpp produced than with Mg^2+^, respectively; Figure 2B,C). A more detailed analysis with Co^2+^ revealed that contrary to Mg^2+^, the optimal concentration for this cation is 8 mM, yielding 1.25 fold more pppGpp than at 16 mM CoCl_2_ (Figure 2A). Under these conditions, RSH_Mex_1-352 produces 2.6 fold more pppGpp than at 8 mM MgCl_2_, and 1.6 fold more than at 16 mM MgCl_2_. Mn^2+^, Ca^2+^, and Ni^2+^ were also tested at 8 mM concentrations, however only in the case of NiCl_2_ higher pppGpp synthesis was observed at 8 mM than at 16 mM concentration, but it was still very low (reaching only 10% of the pppGpp amount synthesized in the presence of 8 mM Co^2+^; Figure 2B,C).

### RSH_Mex_1-352 Has a Much Higher Km for GTP Than ATP

In the next step, we decided to establish RSH_Mex_1-352 kinetics for pppGpp synthesis, namely the apparent Km for ATP and GTP in this reaction. However, first we needed to establish if there would be any pppGpp hydrolysis under our synthesis reaction conditions as this would have a significant bearing on interpretation of obtained results. This was tested by incubating RSH_Mex_1-352 with unlabeled (p)ppNpp nucleotide standards, followed by one-dimensional TLC (Supplementary Figure S3). No enzymatic hydrolysis was observed.

**FIGURE 3:**
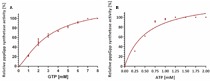
Effects of varying ATP and GTP concentrations on RSH_Mex_1-352 pppGpp synthesis activities in the presence of 8 mM CoCl_2_. **(A)** Apparent Km values for ATP were determined in the presence of 8 mM GTP and varying concentrations of ATP. **(B)** Apparent Km values for GTP were determined in the presence of 8 mM ATP and varying concentrations of GTP. In both cases, each reaction contained 3.3 nM [P^33^] ɣ-ATP, 50 mM Tris–HCl (pH 8.9), and 8 mM CoCl_2_. Relative activity was determined by monitoring pppGpp synthesis by TLC analysis. For each curve, data was normalized to the highest amount of pppGpp obtained. Error bars indicate SD calculated from three independent experiments.

For Km determination, we chose two settings. In one, high GTP concentration (8 mM) was accompanied by ATP titration to determine apparent Km for ATP. In the other, ATP was kept at high concentration and GTP was being titrated. As depicted in Figure 3, RSH_Mex_1-352 has a much higher apparent ability to bind GTP than ATP (apparent Km: 3.0 ± 0.29 mM vs. 0.39 ± 0.01 mM, a 7.8 fold difference).

### RSH_Mex_1-352 Synthesizes pppGpp, ppGpp, and pppApp

Having established optimal reaction conditions for (p)ppGpp, we tested RSH_Mex_1-352 acceptor substrate specificity. Here, RSH_Mex_1-352 was incubated at 37°C for 2 h with 8 mM ATP (pyrophosphate donor) and 8 mM acceptor nucleotides (either ATP, ADP, AMP, GTP, GDP, or GMP). As before, 3.3 nM [P^33^] γ-ATP served as the label, and 8 mM Co^2+^ was employed. Since *S. morookaensis* non-specific pyrophosphotransferase is the only known enzyme to synthesize (p)ppApp, an extract containing this enzyme was used to synthesize (p)ppNpp standards for control reactions that were resolved side by side with the RSH_Mex_1-352 reaction products; these reactions were carried out in the presence of 16 mM MgCl_2_, at 37°C for 15 min.

**FIGURE 4:**
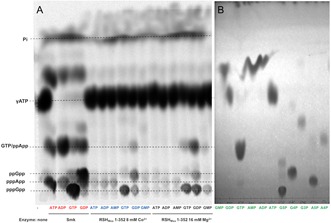
*In vitro* (p)ppNpp synthesis reactions carried out by RSH_Mex_1-352. **(A)** Autoradiogram of a TLC plate with separated (p)ppNpp ’s obtained after synthesis by *S. morookaensis* pyrophosphokinase (**A**, SmK, red) and RSH_Mex_1-352 (**B**, with 8 mM Co^2+^, blue; and **C**, with 16 mM Mg^2+^, black). Substrates used are specified under each lane. The reactions also contained 3.3 nM [P^33^] ɣ- ATP, 8 mM unlabeled ATP, 50 mM Tris–HCl (pH 8.9). To resolve samples on TLC plates, 0.85 M KH_2_PO_4_ (pH 3.4) was used. **(B)** TLC analysis of unlabeled standards (green) separated under the same conditions. G5P, pppGpp; G4P, ppGpp; G3P, pGpp; A5P, pppApp; and A4P, ppApp.

As can be seen in Figure 4, we observed four different RSH_Mex_1-352 reaction products, which could correspond to pppGpp, ppGpp, pppApp, and ppApp. A spot corresponding to pppApp is observed not only in reactions with ATP, but also with ADP and AMP, which is not surprising given those two reactions also contained 8 mM ATP. In reactions with GTP and GDP, both pppGpp and ppGpp were detected. In case of GTP, we could suspect a contamination of the nucleotide preparation with some minor amounts of GDP (through GTP 5′ non-enzymatic hydrolysis), however, presence of pppGpp in the reaction with GDP was puzzling. What is even more surprising, there is also a spot apparently corresponding to ppApp in those two reactions. Similar results were obtained in the presence of 16 mM Mg^2+^, however, in reactions containing ATP and GDP even more of the spot possibly corresponding to ppApp is produced, while the amount of pppGpp produced is roughly equal to that synthesized in a reaction containing ATP and GTP. Still, it should be noted that in this buffer system (0.85 M KH_2_PO_4_, pH 3.4), ppApp co-migrates with GTP as judged by TLC of unlabeled nucleotide standards. Co-migration with some other nucleotide derivatives could not be excluded at this point as well.

**FIGURE 5:**
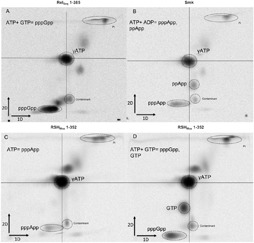
Two-dimensional TLC separation of (p)ppNpp’s synthetized by different enzymes. **(A)** Rel_Seq_1-385 with 8 mM ATP + 8 mM GTP, **(B)** pyrophophokinase from *S. morookaensis* (Smk) with 8 mM ATP + 8 mM GDP, **(C)** RSH_Mex_1-352 with 16 mM ATP, **(D)** RSH_Mex_1-352 with 8 mM ATP + 8 mM GTP. The reaction mixtures contained 3.3 nM [P^33^] ɣ-ATP, 50 mM Tris–HCl (pH 8.9), and either 16 mM MgCl_2_ (Rel_Seq_1-385 and Smk) or 8 mM CoCl_2_ (RSH_Mex_1-352). 1D buffer: 3.3 M ammonium formate + 4.2% boric acid (pH 7), 2D buffer: 0.85 M KH_2_PO_4_ (pH 3.4).

We then pursued nucleotide identification by two-dimensional TLC. The running buffers were chosen so as to distinguish between NTPs and (p)ppNpp derivatives (Supplementary Figure S4; see section “Materials and Methods” for details) (Cashel et al., 1969). The following control reactions were employed: ATP + GTP with Rel_Seq_1-385 (to visualize pppGpp), and ATP + ADP with *S. morookaensis* pyrophosphotransferase (to visualize pppApp and ppApp) (Figure 5A,B). In all cases, [P^33^]-γATP served as the label.

We observed that in the reaction with 16 mM ATP and 8 mM Co^2+^, RSH_Mex_1-352 indeed synthesized pppApp, which to our knowledge is a first documented example of an RSH enzyme synthesizing this nucleotide derivative (Figure 5C). On the other hand, in the reaction with 8 mM ATP and 8 mM GTP, we observed only pppGpp and no pppApp (Figure 5D). We did not observe ppApp (i.e., adenosine tetraphosphate) in any of the reactions catalyzed by RSH_Mex_1-352. However, in the ATP + GTP reaction we detected a spot that corresponds to GTP, as judged by migration of unlabeled nucleotide standards (Supplementary Figure S4). In fact the spot initially ascribed by us to possibly be ppApp is GTP.

We then decided to take another approach that would also validate the observed labeling of GTP by [P^33^]-γATP in another way. A large scale synthesis reaction employing 8 mM ATP, 8 mM GDP, 16 mM MgCl_2_, and RSH_Mex_1-352 was carried out for 24 h, using conditions that gave the highest yield of the nucleotide to be identified. The products were then separated by ion exchange chromatography on Sephadex QAE-25, using LiCl gradient for elution. This was followed by TLC, as well as by measurement of the purified compound’s absorbance in the UV-spectrum. The data obtained confirmed that GTP is indeed produced along with ppGpp, pppGpp and AMP, but not ppApp (Supplementary Figure S5).

### pppApp Is Detected *in vivo* in Wild-Type *M. extorquens* AM1, *B. subtilis*, and *E. coli* Strains

**FIGURE 6:**
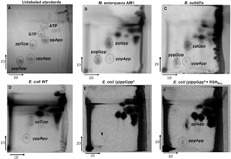
*In vivo* labeling of cellular nucleotide pools with [P^33^]. Nucleotides were labeled, extracted and resolved by TLC as described in the section “Materials and Methods.” **(A)** nucleotide standards, **(B)** *M. extorquens* AM1, **(C)** *B. subtilis*, **(D)** *E. coli*, **(E)** *E. coli* ppGpp^0^ with pUC19, and **(F)** *E. coli* ppGpp^0^ with pUC19-RSH_Mex_. First dimension buffer: 1 M LiCl, 4 M formic acid; second dimension buffer: 0.85 M KH_2_PO_4_ (unadjusted pH).

We take the above experiments as convincing demonstration that pppApp is synthesized *in vitro* by the catalytic domain of RSH_Mex_. A logical next step to establish its biological relevance would be to ask whether pppApp presence is demonstrable *in vivo*. We thus performed *in vivo* [P^33^] labeling of nucleotide pools in bacterial cells. In this case, a different buffer system for 2D TLC was employed than above, so as to better separate all nucleotide pools (Figure 6A) (Nishino et al., 1979). The growth medium used was not limiting for any nutrient, except that low level of inorganic phosphate had to be employed to give [P^33^] specific activities high enough to detect basal (p)ppNpp levels.

As demonstrated by Figure 6B, trace amounts of pppApp and larger amounts of (p)ppGpp are detected in *M. extorquens* AM1 cellular nucleotide extracts. This suggests *in vivo* verification of our *in vitro* data. Importantly, similar results were obtained for *B. subtilis* (Figure 6C), reinforcing previous observations by others (Rhaese et al., 1977; Nishino et al., 1979).

Because of our previous interest in pppApp regulating transcription by *E. coli* RNA polymerase at the *rrnB* P1 promoter, we performed parallel experiments with wild type *E. coli* cells. As can be seen in Figure 6D, quite unexpectedly we were able to observe both – ppGpp and pppApp under our experimental conditions. They are not detected in the *ΔrelA ΔspoT* (ppGpp^0^) strain (Figure 6E). That deficiency is repaired when the ppGpp^0^ strain is transformed with a pUC19 derivative bearing full-length RSH_Mex_, assayed by induction with 0.1 mM IPTG (Figure 6F). There, ppGpp and pppApp are again detected. We ascribe the lack of detectable pppGpp in *E. coli* cells due to the presence of the GppA γ-phosphate hydrolase known to convert pppGpp to ppGpp (Mechold et al., 2013).

### Assays for RSH_Mex_ Regulatory Activity *in vivo*

In the final stage of this work, we tested whether expressing RSH_Mex_ in *E. coli* cells can provoke regulation of the sort predicted from *in vitro* observations with the *rrnB* P1 promoter for (p)ppGpp and pppApp (Bruhn-Olszewska et al., 2018). For this purpose, three different plasmid constructs were used: full length RSH_Mex_, the catalytic domain (RSH_Mex_1-352), and the fragment predicted to have only the synthetase domain. All plasmids were derivatives of pUC19 bearing *rsh_Mex_* gene fragments cloned under the *p_lac_* promoter. The three different strains employed were wild type, *ΔrelA* and ppGpp^0^ and all carried a *rrnB* P1-*lacZ* fusion, whose activity is known to be inhibited by (p)ppGpp (Potrykus et al., 2006).

Growth was carried out in minimal medium supplemented with appropriate amounts of amino acids and carbon source (see section “Materials and Methods” for details) and 0.1 mM IPTG, and the *rrnB* P1-*lacZ* fusion activities were determined by β-galactosidase assays.

**FIGURE 7:**
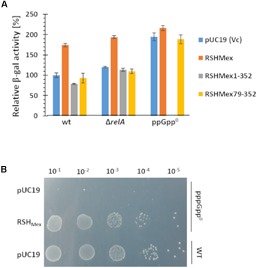
*In vivo* test of effects of (p)ppNpp synthesis. **(A)** RSH_Mex_, RSH_Mex_1-352 (NTD domain), and RSH_Mex_79-352 (only synthetase domain) were cloned into pUC19 and transformed into the indicated host strains carrying the *rrnB* P1-*lacZ* fusion. Growth was carried out in minimal media as described in the section “Materials and Methods,” and β-galactosidase activity was assayed when the cells reached stationary phase. IPTG was added to 0.1 mM. We were unable to obtain ppGpp^0^/RSH_Mex_1-352 transformants. Error bars indicate SD of the values obtained for three independent transformants and their cultures for each strain with the corresponding plasmids. **(B)** growth on SMG plate of the strains indicated, IPTG was added to 0.1 mM.

The results obtained are depicted in Figure 7A. In the case of strains overproducing full length RSH_Mex_ protein, we observed an increase in the *rrnB* P1-*lacZ* fusion activity in the wild type and Δ*relA* strains, roughly reaching the level observed in the ppGpp^0^/vector control strains. There is no substantial increase in activity in case of ppGpp^0^/RSH_Mex_ when compared to the vector control. On the other hand RSH_Mex_1-352 caused a slight decrease (about 25%) in the *rrnB* P1-*lacZ* fusion activity in the wild type strain background, and had no effect in the *ΔrelA* strain when compared to the vector control. Interestingly, we were unable to obtain transformants of the ppGpp^0^ strain with the RSH_Mex_1-352 plasmid.

In addition, we observed that RSH_Mex_79-352, expressing only the predicted synthetase domain of RSH_Mex_, gives the same results as the vector control in all strain backgrounds, meaning this construct is inefficient in (p)ppNpp synthesis without the hydrolase domain, similarly to what was previously observed in Rel_Seq_ studies (Mechold et al., 2002).

Finally, we tested whether RSH_Mex_ could complement *E. coli* ppGpp^0^ phenotype when grown on SMG plates, i.e., minimal glucose plates containing only serine, methionine and glycine as amino acids. This is a growth test of two features. The first is for complementation of the multiple amino acid auxotrophic requirements (DEFHILSTV) of ppGpp^0^ strains because eight of the nine required are missing in the medium (Potrykus et al., 2011; Vinella et al., 2012). The second is for complementation of the stringent response due to an isoleucine deficiency in *E. coli* K-12 strains provoked in minimal medium by the presence of serine, methionine and glycine (Uzan and Danchin, 1976). Figure 7B demonstrates *E. coli* ppGpp^0^/RSH_Mex_ strain complements both, the multiple amino acid requirements of a ppGpp^0^ strain, as well as activation of the isoleucine synthesis (SMG resistance).

## Discussion

This study of the RSH_Mex_ enzyme activity is the first direct biochemical demonstration that an RSH enzyme is capable of synthesizing a nucleotide derivative other than (p)ppGpp, i.e., pppApp. This is shown *in vitro* with the RSH_Mex_1-352 protein (Figure 3, 4), as well as *in vivo* with *E. coli* cells induced to express full-length RSH_Mex_ and with wild type *M. extorquens* AM1 cells (Figure 6). Moreover, we demonstrate pppApp accumulation in wild type *E. coli* cells, which points to a possible new player in bacterial stringent response.

In this work, it became evident to us that there may be a need to re-evaluate RSH nomenclature, changing it from the traditional standard of Rel_(Species-name)_ to RSH_(Species-name)_ and therefore in this case we use RSH_Mex_. We suspect the problem arose when the Cashel-Mechold lab began studies on what they then thought was a *relA*-like monofunctional protein from a *S. equisimilis* strain. This led to calling it Rel*_Seq_* as the RelA protein from *S. equisimilis* (Mechold et al., 1996). It was later discovered to be a bifunctional RSH protein but the name was not changed. The *E. coli* RelA is a monofunctional enzyme capable only of (p)ppGpp synthesis, but not its hydrolysis. We trust that the RSH_Mex_ full-length enzyme is bifunctional and capable of both, (p)ppNpp synthesis and hydrolysis (see below). Also, as already mentioned in the section “Results,” this enzyme has a higher homology to SpoT and Rel_Seq_ (both are bifunctional enzymes) than RelA. There are many obvious future experiments to be done, such as exploring the interaction of RSH_Mex_ with ribosomes, what sources of physiological stress activate synthesis and what responses are mediated by pppApp. This aside, classically enzymes are named for their catalytic properties and/or ortholog or paralog relatedness to other enzymes, rather than on their ability to bind to certain structures (such as ribosomes). Accordingly, RSH for RelA SpoT homologs is meaningful and clear. Thus, we propose that bifunctional homologs should be named RSH, and monofunctional large synthetases termed Rel. Again, the RSH naming does not relate to an ability to bind ribosomes. The abbreviations for small alarmone hydrolases and synthetases, SAH and SAS, respectively, remain clear.

So far, (p)ppApp synthesis has been only demonstrated *in vitro* by a pyrophosphotransferase from *S. morookaensis*, which is a non-specific enzyme transferring pyrophosphate residues from either ATP or GTP onto the ribosyl 3′hydroxyl of any purine nucleotide (Oki et al., 1975). *In vivo, B. subtilis* has been reported long ago to produce (p)ppApp but no biochemical evidence of synthesis was provided (Rhaese et al., 1977; Nishino et al., 1979).

In addition, we also demonstrate that (p)ppGpp synthesis by RSH_Mex_ might entail a non-canonical mechanism. For RelA and Rel_Seq_, as well as several SAS enzymes, it has been shown that (p)ppGpp synthesis is carried out by direct transfer of the γβ-phosphate groups of ATP onto 3′ end of GTP or GDP, to yield pppGpp, or ppGpp respectively (Cashel and Kalbacher, 1970; Mechold et al., 1996, 2002; Hogg et al., 2004; Steinchen et al., 2015). Here, we found that ATP and GTP or GDP gave the expected pppGpp and ppGpp products, in addition to unexpected GTP labeled by the γ-phosphate of ATP (Figure 3, 4 and Supplementary Figure S6). Further studies will reveal whether this GTP molecule is a direct product, an intermediate or perhaps a by-product of the (p)ppGpp synthesis reaction.

Still, it must be noted here, that even though we rigorously identified that additional nucleotide as GTP, and excluded a possibility that the spot identified by us corresponds to other triphosphate guanosine nucleotide derivatives, such as ppGp and pGpp (when run on TLC in 0.85 M KH_2_PO_4_ buffer, these two nucleotides migrate much faster than GTP), it cannot be ruled out completely that this nucleotide may correspond to a yet another, previously unidentified guanosine derivative whose biochemical properties match those of GTP.

Other aspects of the RSH_Mex_1-352 catalyzed reaction are unusual as well. First, we discovered that cobalt cations are required for the most efficient (p)ppNpp synthesis by RSH_Mex_1-352 (Figure 2). For other RSH and SAS enzymes, including RelA, Mg^2+^ gives the most efficient synthesis, although we are not aware of any reports on testing Co^2+^ with these enzymes (for e.g., Mechold et al., 2002; Avarbock et al., 2005; Sajish et al., 2007; Steinchen et al., 2015; Ruwe et al., 2017; Manav et al., 2018). Second, Km values for ATP and GTP reveal RSH_Mex_1-352 has a much higher affinity for ATP than GTP (Figure 5), which is in contrast with observations made with Rel_Seq_ (Mechold et al., 2002).

Still, in contrast to RSH_Mex_ we find Co^2+^ does not support the synthesis of (p)ppGpp by Rel_Seq_ (MS and KP, personal communication). Interestingly, *M. extorquens* AM1 displays cobalt requirement for methylotrophic growth, where it was found that ethylmalonyl-CoA pathway enzymes are cobalt-dependent (Kiefer et al., 2009). Intriguingly, adding CoCl_2_ to the liquid medium for *in vivo* labeling did not enhance pppApp production in our study.

It is noteworthy that under all conditions tested (even with different combinations of Mn^2+^, Mg^2+^, and Co^2+^) we did not observe any of the classical evidence for (p)ppNpp hydrolysis by RSH_Mex_1-352 *in vitro*, namely release of an intact pyrophosphate, as documented for SpoT or Rel_Seq_ (Supplementary Figure S3, and: MS and KP, personal communication), which could mean that this activity might be regulated by the CTD domain, missing in the enzyme we studied *in vitro*. These results seem to be in line with our *in vivo* data where we were unable to obtain transformants of the ppGpp^0^ strain even with an uninduced multicopy plasmid carrying RSH_Mex_1-352, however, transformation was possible with a plasmid bearing the full-length RSH_Mex_. This observation implies that RSH_Mex_1-352 might produce toxically high amounts of (p)ppGpp in this background (Figure 7). SpoT, present in the wild type and *ΔrelA* strains, is able to lower these amounts to the non-toxic levels. In line with these observations, a study was published recently on another representative of alpha-proteobacteria, namely *Caulobacter crescentus* RSH enzyme (called by the authors SpoT) (Ronneau et al., 2018). The authors had shown this enzymes requires the ACT domain (part of the large regulatory C-terminal domain) for ppGpp hydrolysis. Accordingly, this domain is missing in RSH_Mex_1-352, but present in full-length RSH_Mex_.

On the other hand, we observed an increase in the *rrnB* P1-*lacZ* fusion activity for the full-length RSH_Mex_ construct in both – the wt and Δ*relA* backgrounds, while the activity was practically unchanged in the ppGpp^0^ strain when compared to the vector control. A possible explanation is that when SpoT is present, it can hydrolyze RSH_Mex_ – synthesized (p)ppGpp but not pppApp (or not as efficiently as (p)ppGpp). Recently, we found pppApp activates transcription initiating at the *rrnB* P1 promoter *in vitro* (Bruhn-Olszewska et al., 2018), and here the same could be true *in vivo*. Induction of the full length RSH_Mex_ plasmid in the wild type or *relA* mutant hosts does significantly elevate *rrnB* P1reporter activity, as if pppApp might overcome the negative regulatory effects of low levels of ppGpp to parallel pppApp activities observed *in vitro* (Bruhn-Olszewska et al., 2018). Lack of increase in the *rrnB* P1-*lacZ* fusion activity in the ppGpp^0^/RSH_Mex_ strain could be due to the maximum promoter activity already achieved in the ppGpp^0^ background, which simply cannot be increased any further.

Also, Figure 6F shows that in the IPTG induced ppGpp^0^/RSH_Mex_ strain both ppGpp and pppApp are clearly present. On the basis of ppGpp levels alone, quite strong inhibition of *rrnB* P1 might be predicted but is not seen. This again implies that activation by pppApp occurs, and despite its low abundance relative to ppGpp it can overcome the (p)ppGpp mediated inhibition of the *rrnB* P1 promoter. On the other hand, stable survival of this strain again suggests RSH_Mex_ -mediated hydrolysis, since unchecked production of (p)ppNpp would be toxic to the cell, as demonstrated by inability to transform ppGpp^0^ strain with RSH_Mex_1-352. This, together with ppGpp^0^/RSH_Mex_ SMG plate growth (Figure 7), suggest that instead of pppApp effects, perhaps RSH_Mex_-mediated ppGpp synthesis at low levels could be enough to induce isoleucine synthesis and high enough to complement the multiple amino acid requirements of ppGpp^0^ strains, as noted earlier with [P^33^] labeled nucleotide extracts (Figure 6).

Overall, our *rrnB* P1-*lacZ* reporter studies hint that pppApp might display positive regulatory effects *in vivo*. Rigorous evidence awaits devising a means of selectively manipulating incremental cellular accumulation of pppApp independently of ppGpp abundance so that competitive regulation can be assessed, much like regulatory potency for ppGpp vs. pppGpp has been investigated (Mechold et al., 2013).

Might pppApp accumulation be found among diverse bacterial species and participates in stress responses along with (p)ppGpp? It is intriguing that we observed cellular accumulations of pppApp but not ppApp, since *in vitro* transcription studies reveal pppApp has a much stronger effect on RNA polymerase than ppApp (Bruhn-Olszewska et al., 2018). If RNA polymerase regulatory effects of pppApp are generally present in bacteria then it is predicted that *E. coli* RNAP residues contacting pppApp (Bruhn-Olszewska et al., 2018) might also be found in RNA polymerases of *B. subtilis* and *M. extorquens*. Such comparisons do reveal conservation among these species with respect to residues R346, R352, A426, and Q465 of the β′ subunit, and K1242 of the β subunit. It is an especially intriguing finding for *B. subtilis* RNAP because in this organism, control of rRNA synthesis by (p)ppGpp is clearly known to occur indirectly through control of GTP levels rather than direct interactions of (p)ppGpp with RNAP (Krasny and Gourse, 2004).

Since pppApp co-migrates with ppGpp on TLC plates in commonly used assays employing 1 M KH_2_PO_4_ (pH 3.4) resolution buffer (Supplementary Figure S3), the question arises as to whether previous measurements of ppGpp in different bacteria were in fact distorted by unsuspected pppApp content. It is likely that instances will be found where pppApp was mistakenly included in quantitation with ppGpp by TLC resolution or dismissed as contamination. On the other hand, neither our current work nor that of others (Rhaese et al., 1977; Nishino et al., 1979) have detected cellular (p)ppApp at high levels, e.g., equal to or exceeding GTP, as commonly found for (p)ppGpp. Therefore future studies should have priority for finding conditions where (p)ppApp production levels increase and establishing the mechanism of its synthesis *in vivo*. In addition an effort should focus on developing better TLC methods to resolving all four nucleotides pppApp, ppGpp, ppApp, and GTP.

## Materials and Methods

### Strains and Plasmids

All strains used, plasmids and their construction are described in Supplementary Table S1.

### Protein Purification

For *in vitro* studies, Hisx8-SUMO-tagged proteins (RSH_Mex_1-352 and Rel_Seq_1-385) were purified by Ni^2+^-NTA affinity chromatography, followed by removal of the His-SUMO tag by digestion with Ulp1 SUMO protease. In detail, *E. coli BL21 (DE3) Rosetta* cells carrying a plasmid for His-SUMO-tagged protein production were grown at 30°C in 2 L of LB (0.5% NaCl), supplemented with kanamycin at 50 μg/ml. Cells were cultivated to OD_600_ = 0.6. Expression was induced by adding IPTG to the final concentration of 0.4 mM, and cultivation was continued overnight at 16°C. Cells were harvested by centrifugation, re-suspended in 100 ml of lysis buffer (20 mM of Tris–HCl pH 8.0, 500 mM NaCl, 20 mM imidazole, 10% glycerol, 2 mM β-mercapthoethanol), PMSF was added, and the cells were lysed by sonication. After centrifugation (37,850 × *g*, 30 min, 4°C), clear supernatant was loaded on a BioRad disposable column pre-loaded with 2 ml of the Ni-NTA Superflow resin (Thermo Scientific) and pre-equilibrated with 10 ml of lysis buffer but without imidazole. Following a wash with 40 ml of lysis buffer, the His-SUMO-tagged protein was eluted with 10 ml of elution buffer (lysis buffer but with 180 mM imidazole). Protein containing fractions (10 ml) were pooled and dialyzed against 2 L of dialysis buffer (20 mM of Tris–HCl pH 8.0, 250 mM NaCl, 10% glycerol) in a Slide-a-lyzer 10K cassette (Thermo Scientific). His-SUMO-tag was cleaved by an in-house purified His-tagged yeast Ulp1 SUMO protease, added to the final concentration of 10 μg/ml, and incubated at 4°C for 15 min. After cleavage, the protein sample was loaded on a new BioRad column pre-loaded with 2 ml of Ni-NTA Superflow resin, pre-equilibrated with 10 ml of dialysis buffer. Flow through fraction obtained this way contained pure, untagged proteins. Glycerol was added to the final concentration of 50% and preps were stored at -80°C. Protein concentration was determined with Qubit 2.0 (Thermo Scientific), and purity was determined by SDS-PAGE. This protocol yielded 10 ml of RSH_Mex_1-352 at a final concentration of 0.186 mg/ml, with >99% purity (Supplementary Figure S6). For Rel_Seq_1-385, 10 ml of 13.6 mg/ml protein were obtained (with 98% purity).

The *S. morookaensis* excreted ammonium sulfate precipitated protein extract containing the non-specific pyrophosphotransferase was prepared as described in (Bruhn-Olszewska et al., 2018).

### Nucleotides and Unlabeled (p)ppNpp Standards

All NTPs, NDPs, and NMPs used were purchased from Sigma-Aldrich. Unlabeled (p)ppNpp standards were prepared and purified as described in (Bruhn-Olszewska et al., 2018).

### *In vitro* Preparation of P^33^-Labeled Nucleotides for Their Identification and for Optimal Reaction Conditions’ Assessment

For RSH_Mex_1-352, *in vitro* (p)ppNpp synthesis was generally carried out with either 80 or 160 nM of purified protein in a reaction containing 50 mM Tris–HCl pH 8.9, 3.3 nM [P^33^] γ-ATP (Perkin Elmer), and 8 mM ATP (pyrophosphate donor) and 8 mM acceptor nucleotide (ATP, ADP, AMP, GTP, GDP, or GMP). The cation concentrations used were: 8 mM Co^2+^ or 16 mM Mg^2+^ (unless indicated otherwise), provided as CoCl_2_ or MgCl_2_. MnCl_2_, CaCl_2_, or NiCl_2_ were also tested. The reactions were carried out at 37°C, and the reaction time varied between 1 and 16 h. Reactions were stopped by adding an equal volume of 2 M formic acid and spotted onto 20 cm × 20 cm PEI-Cellulose plates (Merck). One-dimensional thin layer chromatography (TLC) was carried out in either 0.85 or 1 M KH_2_PO_4_ (pH 3.4) buffer. For two-dimensional TLC, samples were first separated in a buffer containing 3.3 M ammonium formate and 4.2% boric acid (pH 7.0); this was followed by soaking plates for 15 min in methanol, rinsing in water, development in the second dimension in 0.85 M KH_2_PO_4_ (pH 3.4). Autoradiograms were visualized using a phosphorimager (Typhoon 9200, GE Healthcare).

For the Rel_Seq_1-385 and *S. morookaensis* pyrophosphotransferase containing extract, similar reaction conditions were used as above, with 16 mM MglCl_2_ and 160 nM Rel_Seq_1-385 protein or 2 μl of the S. *morookaensis* extract. Reactions were carried out for 30 min at 37°C.

### RSH_Mex_1-352 Enzyme Kinetics

Measurements of RSH_Mex_1-352 enzyme kinetics were carried out as described above but with 80 nM protein. Reactions were carried out with 8 mM CoCl_2_ for 2 h. End-point assays were used since using continuous assays was impractical in this case due to low method sensitivity in detecting low (p)ppNpp amounts. The values obtained should be taken with caution and thus are referred to as apparent Km. The apparent Km for GTP was measured with ATP at 8 mM and titrated GTP (1–8 mM). For the apparent Km for ATP, GTP was held at 8 mM and ATP varied from 0.25 to 2 mM. The samples were processed as described above and TLC was run in 1.5 M KH_2_PO_4_ (pH 3.4) buffer. Autoradiograms were visualized using a phosphorimager (Typhoon 9200, GE Healthcare). Spots corresponding to pppGpp were quantitated using a UVP Visionworks software and the data was analyzed with GraphPad Prism 5; apparent Km values were obtained from the fit to the following equation: V = [Vmax (S)] ÷ [Km + (S)].

### Preparation of [P^33^]- Labeled Cellular Nucleotide Extracts

*Methylobacterium extorquens* AM1 was cultivated on LB plates supplemented with 0.1% meat extract (Sigma), 0.1% methanol and rifampicin (50 μg/ml). *E. coli* and *B. subtilis* strains were grown on LB plates. Bacteria were scraped off plates and resuspended in the Tris-glucose medium (0.1 M Tris pH 7.4, 0.1 mM KH_2_PO_4_, sodium citrate (0.42 mg/ml), MgSO_4_ × 7H_2_0 (0.21 mg/ml), (NH_4_)_2_SO_4_ (1 mg/ml), FeCl_3_ (0.32 μg/ml), glucose (0.2%), and the following amino acids: lysine, proline, glycine, alanine, glutamic acid, aspartic acid, arginine, at 100 μg/ml; cysteine, methionine, tyrosine, tryptophan, and phenylalanine at 40 μg/ml) (Nishino et al., 1979). Nucleotides were labeled by the addition of [P^33^] – phosphoric acid to 5 μCi/ml and incubated with shaking at 30°C for 45 min. For *E. coli* strains carrying pUC19 derivatives, 0.1 mM IPTG was added. Reactions were stopped by the addition of an equal volume of 23.6 M formic acid, and followed by three freeze-thaw cycles in liquid nitrogen. Extracts were centrifuged (14,000 × *g*, 10 min, room temperature) and the supernatants were spotted onto PEI-Cellulose plates. For two-dimensional TLC separation, first dimension buffer contained 1 M LiCl and 4 M formic acid; this was followed by soaking plates for 15 min in methanol and a second dimension run in 0.85 M KH_2_PO_4_ (unadjusted pH). Autoradiograms were visualized as before.

### *rrnB* P1-*lacZ* Fusion Assay and ppGpp^0^ Phenotype Complementation

Appropriate *E. coli* strains carrying *rrnB* P1-*lacZ* chromosomal fusions were transformed with pUC19-derived plasmids carrying genes of interest and streaked on minimal medium plates containing: 1 × M9 salts (BioShop), 1.5% agar, 1% vitamin B1, 0.5 μM FeSO_4_, 0.2% casamino acids, 0.02% glucose, and 0.1 mM IPTG. Ampicillin was added to 50 μg/ml. Plates were incubated at 30°C for 48 h. The cells were then inoculated into the same medium (but lacking agar), grown with shaking to stationary phase, and β-galactosidase activity was assayed as described in (Miller, 1972).

For *E. coli* ppGpp^0^ complementation assay, cells were collected from plates, washed three times with 0.9% NaCl, resuspended in 0.9% NaCl to adjust OD_600_ to 0.1, and 5 μl of appropriate dilutions were spotted on SMG plates (minimal medium plates as above but with 100 μg/ml each of serine, methionine and glycine instead of casamino acids). Growth was carried out as described above.

## Author Contributions

KP conceived this study. KP, MS, and MC designed the experiments and MS and KP performed them. BB-O participated in GTP identification. KP, MS, and MC analyzed the data. KP and MC wrote the main text. All authors discussed the results, commented on the manuscript, and approved its final version.

## Conflict of Interest Statement

The authors declare that the research was conducted in the absence of any commercial or financial relationships that could be construed as a potential conflict of interest.
