# Supplementary material for: Methylobacterium extorquens RSH Enzyme Synthesizes (p)ppGpp and pppApp in vitro and in vivo, and Leads to Discovery of pppApp Synthesis in Escherichia coli
Source: Front Microbiol. 2019 Apr 24;10:859. doi: 10.3389/fmicb.2019.00859 (PMC6491832; doi:10.3389/fmicb.2019.00859)
Supplement: Supplementary file 1 [file Data_Sheet_1.pdf]

Supplementary Material

Table S1. Strains and plasmids used in this study.

**Strains**		
Name	Genotype	Source
Bacillus subtilis 168	wild type	Kunst et al., 1997
Methylobacterium extorquens AM1	wild type	Nunn and Lidstrom, 1986
Escherichia coli		
BL21 DE3 Rosetta	F- *ompT gal dcm lon hsdSB*(*rB-* *mB*-) λ(DE3)/ pRARE (Cam^R)	Novagen®
CF1648	K-12, F – λ– *ilvG– rfb-50 rph-1*	Xiao et al., 1991
CF6301	CF1648 *ΔrelA:kan rrn*B P1’::*lacZ-kan*	Vinella et al., 2000
CF6306	CF6301 (*ΔrelA ΔspoT*) *ΔlacZ(mluI) ΔspoT207::cat rrnB* P1’::*lacZ-kan*	Potrykus et al., 2006
CF7753	CF1648 *ΔlacZ(mluI) rrn*B P1’::*lacZ-kan*	Cashel lab strain collection
**Plasmids**		
Name	Description	Source
pCIOX	pET-derived plasmid with N-terminal his-SUMO-tag followed by *BamHI*/*Xho*I insertion site; Kan^R	a gift from Dr. Andrea Mattevi; Addgene plasmid # 51300
pMS9	RSH$_{Mex}$1-352 in pCIOX	This work.<br><br>A synthetic DNA fragment encoding putative RSH enzyme from *Methylobacterium extorquens* AM1 (ACS41145.1) has been ordered from Thermo Scientific (GeneArt- GeneStrings). Codon usage and GC % were optimized for efficient overexpression in the *E. coli* host with

		the GeneOptimizer software (Thermo). Catalytic fragment, carrying only the hydrolase and synthetase domains [NTD; corresponding to a protein spanning 1-352 amino acids (RSH_{Mex}1-352)], was obtained by PCR amplification with the following primers:<br><br>MS34:<br>AAGGATCCATGATGCGTCAGTATGAACTGGTTG<br>MS36:<br>5'AACTCGAGTAAAACGCCTTCTTTACCTGCTT3'<br><br>After restriction digestion with *BamH*I/*Xho*I, this fragment was inserted into corresponding sites of pCIOX, in frame with N-terminal Hisx8-SUMO tag.
pMS10	Rel_{Seq}1-385 - pCIOX	This work.<br><br>This plasmid was constructed by PCR amplification of a DNA fragment corresponding to 1-385 aa of Rel_{Seq}; pUM66 served as a template. The following primers were used:<br><br>KPr89:<br>5'ACAGAGAACAGATTGGTGGTCGCGGATCCATGGCAAAAGAAATCAATTTAACA3'<br>KPr90:<br>5'GATGCAGTGGACTTTGTGGACTCGTAAGCTCGAGAGACTAGCTTAGGTATTTAT3'
pMS13	RSH_{Mex}1-744 – pUC19	This work.<br><br>DNA fragment corresponding to 1-744 aa of RSH_{Mex} was amplified based on the same synthetic DNA fragment as was used for pMS9 construction, except that the following primers were used:<br><br>MS42:<br>5'ATACCCGGGTAACCATTAACACGTTCCACACG3'<br>MS45:<br>5'ATAAAGCTTATGATGCGTCAGTATGAACTGGTTG3'

		After PCR amplification, the DNA fragment was digested with *Hind*III/*Sma*I and cloned under p*lac* by insertion into appropriate pUC19 sites.
pMS14	RSH_{Mex}1-352 – pUC19	This work.<br><br>RSH_{Mex} catalytic domain (NTD) containing fragment was cloned into pUC19 in the same way as pMS13, except that the following primers were used:<br><br>MS43<br>5’ATACCCGGGTAAAACGCCTTCTTTACCTGCTT3’<br>MS45:<br>5’ATAAAGCTTATGATGCGTCAGTATGAACTGGTTG3’
pMS15	RSH_{Mex}79-352 – pUC19	This work.<br><br>RSH_{Mex} synthetase only fragment was cloned into pUC19 in the same way as pMS13, except that the following primers were used:<br><br>MS43:<br>5’ATACCCGGGTAAAACGCCTTCTTTACCTGCTT3’<br>MS73:<br>5’ATAAAGCTTATGACCGTTGAAGATACCGCAGC3’
pUC19	p*lac*$_{UV5}$ followed by MCS linker and *lacZ*’; Ap^R	Norrander et al., 1983
pUM66	pBAD18; RelSeq (aa 1–385); C-terminal His tag	Mechold et al., 2002

References:

Kunst F, Ogasawara N, Moszer I, Albertini AM, Alloni G, et al., (1997) The complete genome sequence of the gram-positive bacterium *Bacillus subtilis*. *Nature* 390:249-56.

Mechold U, Murphy H, Brown L, Cashel M. (2002) Intramolecular regulation of the opposing (p)ppGpp catalytic activities of Rel(Seq), the Rel/Spo enzyme from *Streptococcus equisimilis*. *J Bacteriol.* 184:2878-88.

Norrander J, Kempe T, Messing J. (1983) Construction of improved M13 vectors using oligodeoxynucleotide-directed mutagenesis. *Gene* 26:101-6.

Nunn DN, Lidstrom ME. (1986) Isolation and complementation analysis of 10 methanol oxidation mutant classes and identification of the methanol dehydrogenase structural gene of *Methylobacterium sp. strain AM1*. *J Bacteriol.* 166:581-90.

Potrykus K, Vinella D, Murphy H, Szalewska-Palasz A, D'Ari R, Cashel M. (2006) Antagonistic regulation of *Escherichia coli* ribosomal RNA *rrnB* P1 promoter activity by GreA and DksA. *J Biol Chem.* 281:15238-48.

Vinella D, Cashel M, D'Ari R. (2000) Selected amplification of the cell division genes ftsQ-ftsA-ftsZ in *Escherichia coli*. *Genetics.* 156:1483-92.

Xiao H, Kalman M, Ikehara K, Zemel S, Glaser G, Cashel M. (1991) Residual guanosine 3',5'-bispyrophosphate synthetic activity of *relA* null mutants can be eliminated by *spoT* null mutations. *J Biol Chem.* 266:5980-90.

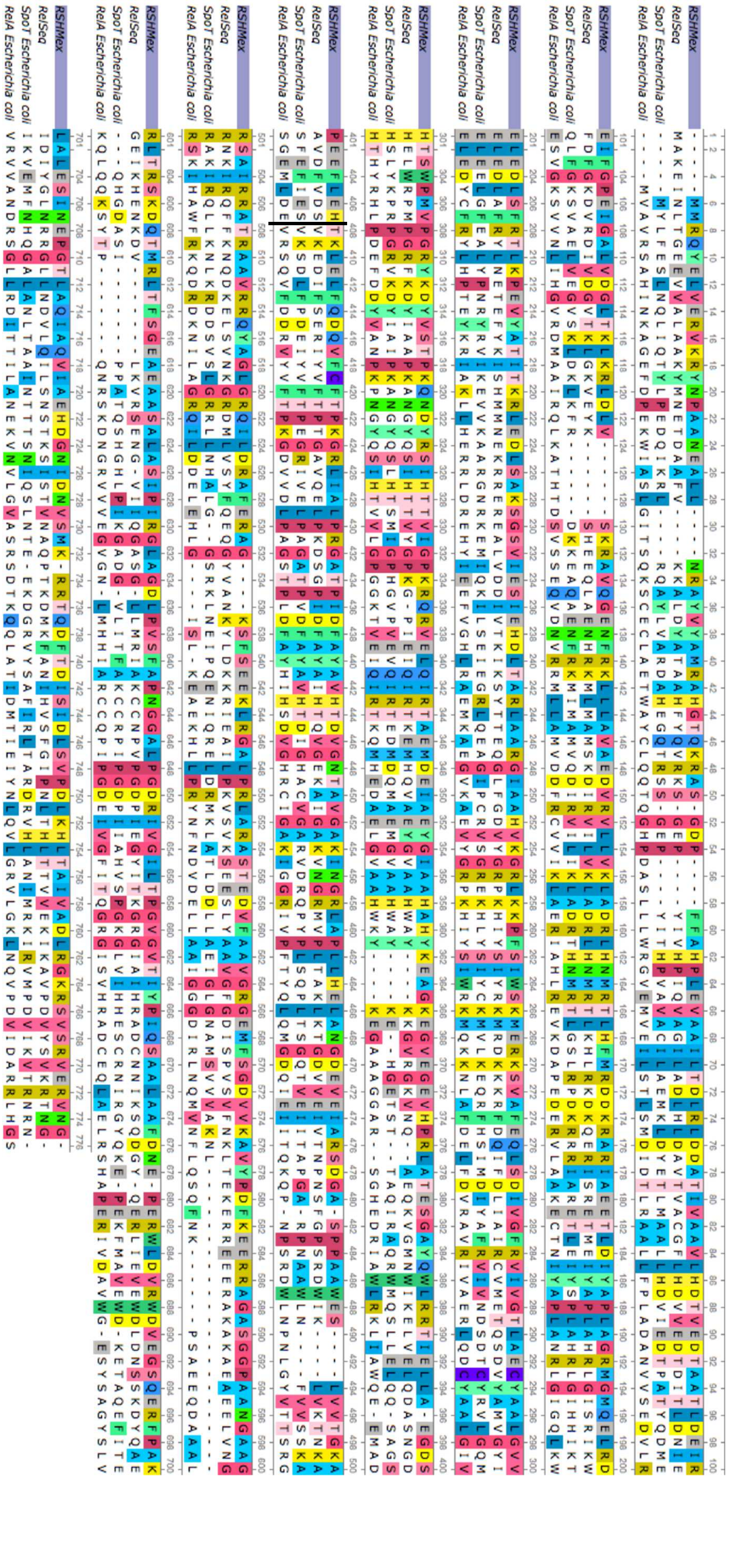

Fig. S1.

Figure S1. Amino acid sequence comparison of RSH_{Mex}, Rel_{Seq}, E. coli RelA and SpoT with the use of UGENE software and MUSCLE algorithm. RSH_{Mex} was used as the reference sequence. Thick black line denotes the end of the NTD catalytic domain, corresponding to RelSeq1-385 and RSH_{Mex}1-352. Full-length RSH_{Mex} has only 38%, 32% and 39% identity with Rel_{Seq}, *E. coli* RelA and SpoT, respectively. In comparison, RelA vs SpoT = 33% , RelA vs Rel_{Seq}= 36% , SpoT vs Rel_{Seq} = 43% .

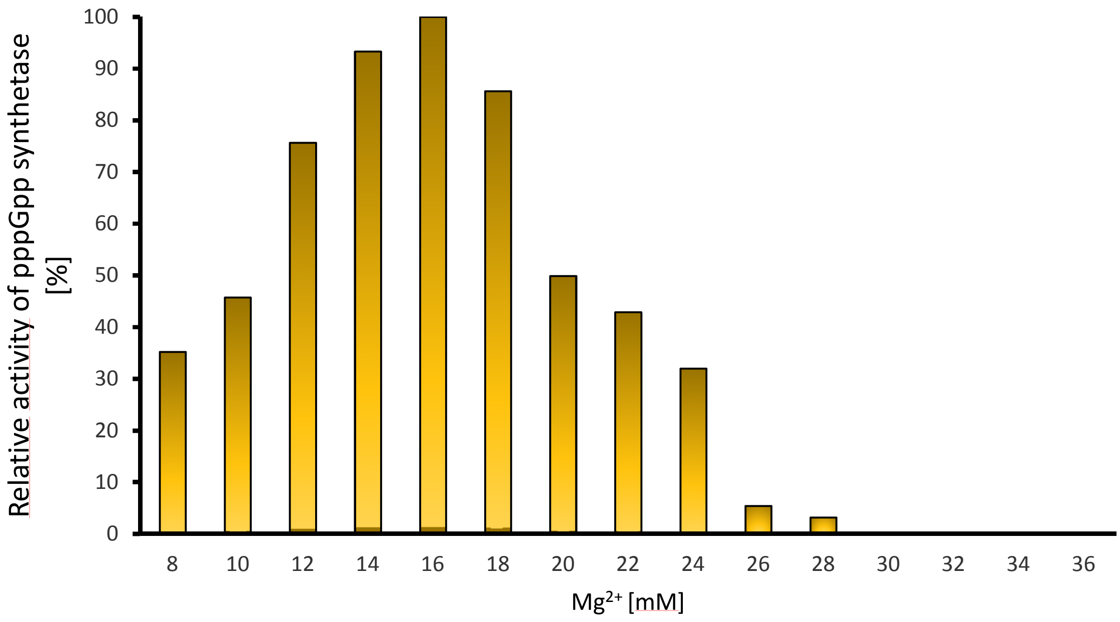

Figure S2. RSH$_{Mex}$1-352 pppGpp synthesis activity in response to MgCl$_2$ titration (8-36 mM). The reaction mixtures contained 3.3 nM [P33] Ɣ-ATP, 8 mM unlabeled ATP, 8 mM GTP, 50 mM Tris-HCl (pH 8.9), 80 nM RSH$_{Mex}$1-352, and were incubated for 2 hrs at 37°C. Relative amount of synthesized pppGpp was determined by TLC followed by densitometry.

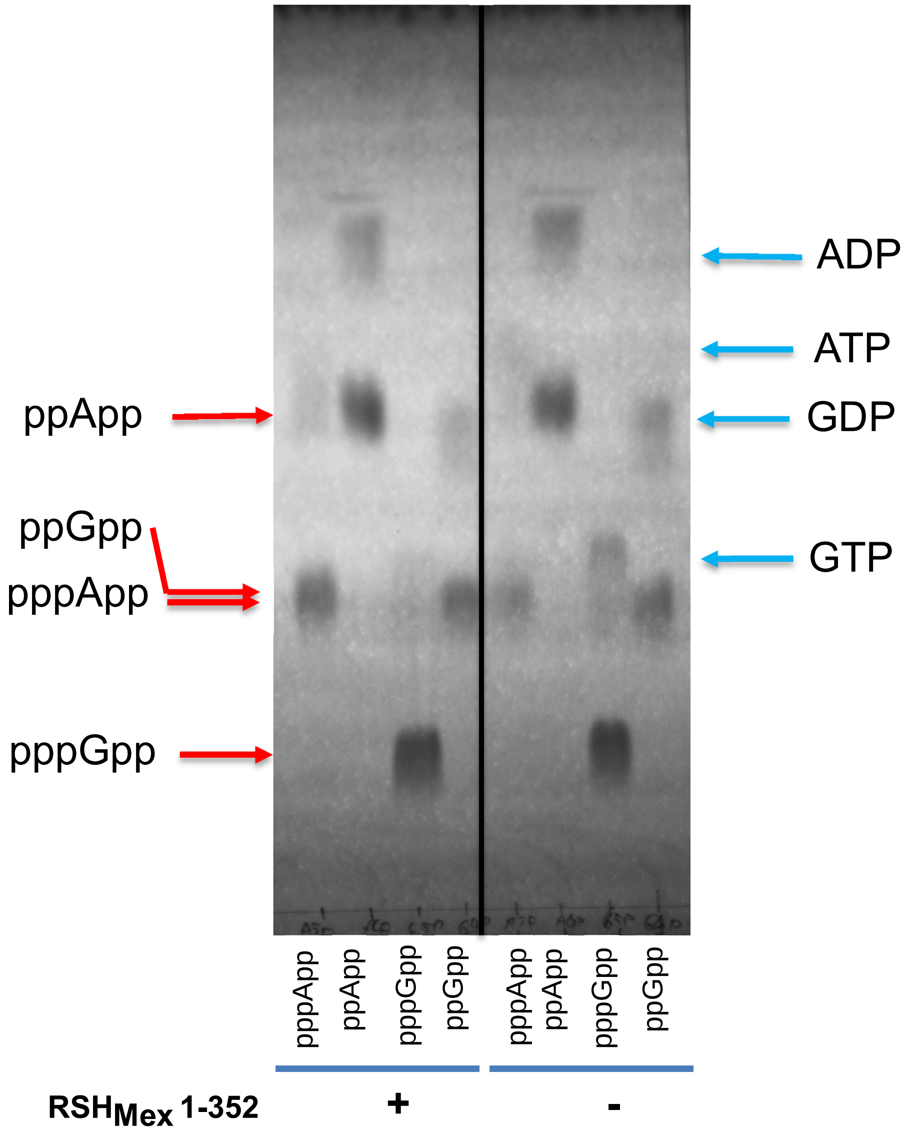

Figure S3. (p)ppNpp hydrolysis by RSH$_{Mex}$1-352. Unlabeled (p)ppNpp standards were used. Reactions contained 8 mM Co2+ and RSH$_{Mex}$1-352 at 160 nM (left panel) or no enzyme (control; right panel). Incubation was carried for 2 hrs at 37°C. Red arrows – (p)ppNpp standards. Blue arrows – nucleotides that arose due to non-enzymatic hydrolysis of (p)ppNpp. Enzymatic hydrolysis has not been observed. TLC plate was resolved in 1 M KH_2PO_4 (pH 3.4).

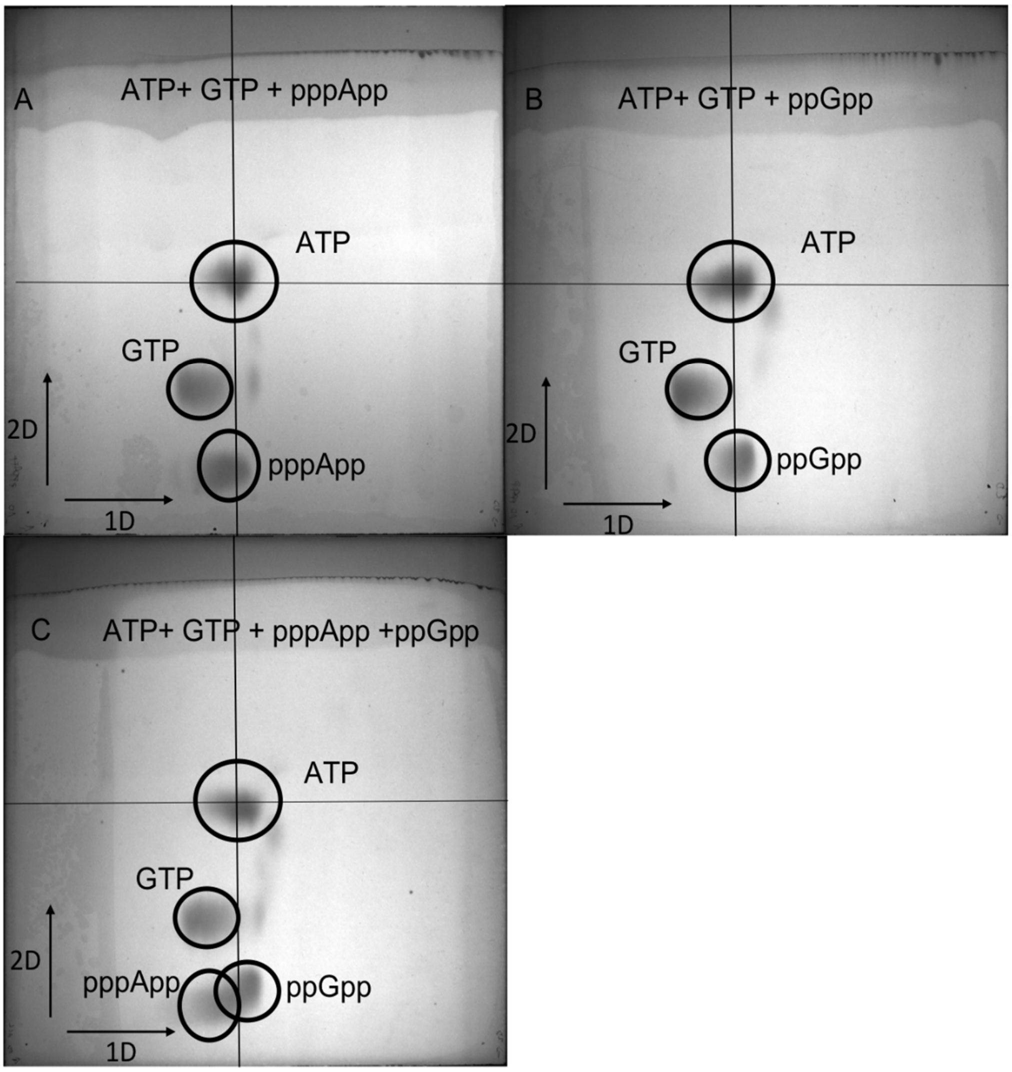

Figure S4. Two-dimensional TLC separation of unlabeled nucleotides. Nucleotides used are specified on each panel. 1D buffer: 3.3 M ammonium formate + 4.2% boric acid (pH 7), 2D buffer: 0.85 M KH_2PO_4 (pH 3.4).

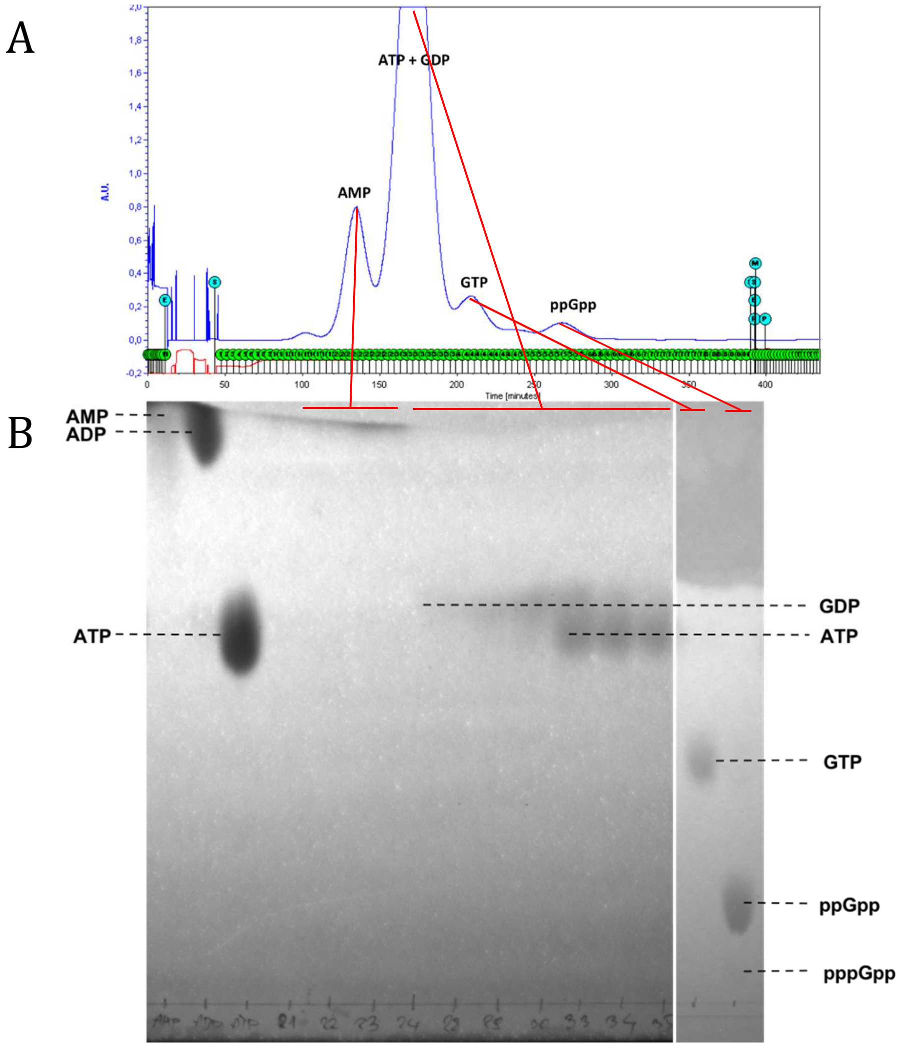

Figure S5. Identification of GTP as a product of RSH_{Mex}1-352 ppGpp synthesis reaction. Large-scale reaction was set up with ATP and GDP as substrates. The reaction mixture contained 50 mM Tris-HCl (pH 8.9), 16 mM $MgCl_2$, 8 mM ATP and 8 mM GDP, as well as 0.5 mg of RSH_{Mex}1-352, and was incubated for 24 h at 37°C. Reaction volume was 5 ml. A) elution profile of ion exchange chromatography on a 18 ml Sephadex QAE-25 column, linear elution gradient was used (buffer A: 0.2 M LiCl, 25 mM Tris pH 8.0, 0.5 mM EDTA; buffer B: 0.4 M LiCl, 0.25 mM Tris pH 8.0, 0.5 mM EDTA, linear gradient volume: 170 ml), BioLogic system (BioRad) was used. B) thin layer chromatography of nucleotides from appropriate fractions obtained in A, run along with ATP, ADP and AMP standards; 0.85 M KH_2PO_4 (pH 3.4) was used.

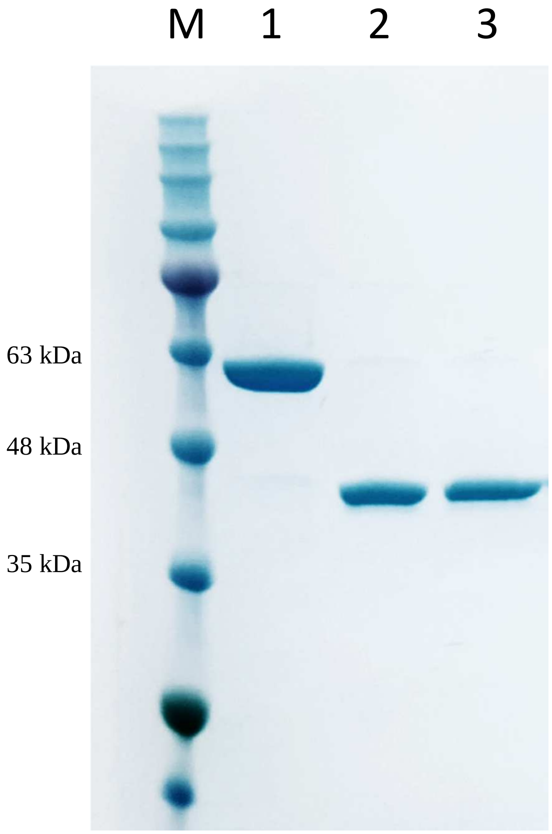

Figure S6. SDS-PAGE of the purified RSH_{Mex}1-352 protein (NTD domain of RSH_{Mex}). Protein purification was carried out as described in Materials and Methods (main manuscript text). Lanes: M - protein ladder (Tricolor, Eurx), 1 – purified SUMO-RSH_{Mex}1-352 before protease treatment (after dialysis), 2 – sample from lane 1 after Ulp1 protease treatment, 3 - stock protein used in this study. Samples were run on a Tris-Gly 4 -12% gradient gel (Thermo).
